# 肺部微生物群与肺癌关系的研究进展

**DOI:** 10.3779/j.issn.1009-3419.2021.102.53

**Published:** 2022-01-20

**Authors:** 征 苏, 鑫华 贾, 亚光 范, 方辉 赵, 友林 乔

**Affiliations:** 1 100021 北京，国家癌症中心，国家肿瘤临床医学研究中心，中国医学科学院北京协和医学院肿瘤医院，流行病学研究室 Department of Epidemiology, National Cancer Center/National Clinical Research Center for Cancer/Cancer Hospital, Chinese Academy of Medical Sciences and Peking Union Medical College, Beijing 100021, China; 2 361102 厦门，厦门大学公共卫生学院国家传染病诊断试剂与疫苗工程技术研究中心 The State Key Laboratory of Molecular Vaccinology and Molecular Diagnostics, National Institute of Diagnostics and Vaccine Development in Infectious Diseases, School of Public Health, Xiamen University, Xiamen 361102, China; 3 300052 天津，天津医科大学总医院，天津市肺癌研究所，天津市肺癌转移与肿瘤微环境重点实验室 Tianjin Key Laboratory of Lung Cancer Metastasis and Tumor Microenvironment, Tianjin Lung Cancer Institute, Tianjin Medical University General Hospital, Tianjin 300052, China; 4 100021 北京，中国医学科学院/北京协和医学院群医学及公共卫生学院 Center for Global Health, School of Population Medicine and Public Health Chinese Academy of Medical Sciences & Peking Union Medical College, Beijing 100021, China

**Keywords:** 肺肿瘤, 肺部微生物群, 致癌机制, 免疫治疗, Lung neoplasms, Lung microbiome, Carcinogenesis, Immunotherapy

## Abstract

微生物群在人体生物学功能中发挥着重要作用，与炎症（胃炎、肝炎）、癌症（胃癌、宫颈癌、肝癌）等多种疾病状态有关。人类微生物组计划在第一阶段描绘了人体微生物的全景图，纳入鼻腔、口腔、肠道、阴道和皮肤等人体部位，而肺部被认为是无菌环境。但近些年研究证实肺部存在丰富的微生物群落，该肺部微生物群与肺部疾病的关联成为研究热点。目前研究发现，与健康人或肺部疾病患者相比，肺癌患者具有特异的微生物群。即使在肺癌患者体内，肿瘤部位也存在特有的肺部微生物群。此外，不同病理类型和转移状态的肺癌也会导致微生物群的差异。机制学研究发现，肺部微生物群可能通过影响免疫反应影响肺癌的发生。目前肺部微生物与免疫治疗的临床研究仍处于初步阶段。未来需要更多相关研究来提供高质量的证据，进一步了解肺部微生物群的致癌机制，为临床治疗提供新思路。本文对肺部微生物群与肺癌的相关性研究，可能的分子机制和临床治疗中的应用，简要综述了肺部微生物群研究进展，并对今后研究提出展望。

## Abstract (en)

The microbiota plays an important role in the biological functions of the human body and is associated with various disease states such as inflammation (gastritis, hepatitis) and cancer (stomach, cervical, liver). The Human Microbiome Project painted a panorama of human microorganisms in its first phase, incorporating body parts such as the nasal cavity, oral cavity, intestine, vagina and skin, while the lungs were considered a sterile environment. However, studies in recent years have confirmed the presence of a rich microbial community in the lung, and the association of this lung microbiota with lung disease has become a hot topic of research. Current research has found that patients with lung cancer have a specific microbiota compared to healthy individuals or patients with lung disease. Even in patients with lung cancer, a lung microbiota specific to the tumor site is present. In addition, different pathological types and metastatic status of lung cancer can lead to differences in microbiota. Mechanistic studies have found that the lung microbiota may influence lung cancer development by affecting the immune response. Clinical studies on lung microbiota and immunotherapy are still in the preliminary stage. More relevant studies are needed in the future to provide high-quality evidence to further understand the oncogenic mechanisms of lung microbiota and provide new ideas for clinical treatment. This paper briefly reviews the progress of lung microbiota research in terms of its relevance to lung cancer, possible molecular mechanisms and applications in clinical treatment, and provides an outlook for future research.

人体微生物群是由细菌、真菌、古细菌、原生动物和病毒等多种微生物组成，与宿主共同进化，其在人体的各种生物学功能中发挥着重要作用^[1, 2]^。微生物群由大约40万亿个微生物组成，数量远超过人类细胞的数量，其特性和相对丰度会受到人体健康状态的影响^[3]^。例如，幽门螺杆菌（*Helicobacter pylori*, HP）是一种常见的蛋白质细菌，存在于上消化道，主要导致胃炎和胃溃疡的发生，并且可以显著增加胃癌的患病风险^[4]^。人乳头状瘤病毒（human papillomavirus, HPV）是一组环状双链DNA（deoxyribonucleic acid）病毒，易感染人类表皮和黏膜鳞状上皮，引起生殖器疣、宫颈癌和口咽癌等多种疾病^[5]^。人乙型肝炎病毒（human hepatitis B virus, HBV）是一种DNA病毒，属于嗜肝DNA病毒科，HBV慢性感染会导致慢性肝炎、肝硬化和肝细胞癌等疾病的发生^[6]^。随着二代测序技术（next generation sequencing, NGS）的发展，人类微生物群已受到广泛关注，微生物群可以通过免疫、代谢和炎症等多个生物途径对人类机体健康状态和疾病治疗等方面产生重要影响^[7]^。

人类微生物组计划（Human Microbiome Project, HMP）于2007年被提出，分为2个阶段。第一阶段主要以健康人群为对象描绘了人体微生物的全景图。第二阶段的人类微生物组计划作为第一阶段的延续，联合多组学研究策略探究微生物在健康与疾病中扮演的角色。在第一阶段，研究纳入的人体部位包括鼻腔、口腔、肠道、阴道和皮肤，而肺部则被认为是无菌的。但近些年研究发现，肺部存在微生物群落并且与肺癌的发生发展有关。研究人员分析肺部呼吸样本中的微生物组群落发现，拟杆菌属、厚壁菌属、变形杆菌属和链球菌属、假单胞菌属、维氏菌属和普氏菌属^[8-11]^等微生物可能与肺癌发病有关。因此，本文中简要综述了近年来肺部微生物群研究进展，包括目前肺部微生物群与肺癌的相关性研究，可能的分子机制和临床治疗中的应用。

## 1 肺部微生物群与肺癌

肺部的微生物群是处于动态平衡状态的，因为呼吸道不断暴露于悬浮在空气中的微生物，这些微生物通过口腔和上呼吸道流向下呼吸道^[12]^。下呼吸道的微生物群通过呼吸和咽部分泌物的微吸入进入肺部，这大概是健康人体肺部微生物群的主要来源^[13]^。考虑到口腔和呼吸道之间的解剖学关联，健康成年人口咽部微生物群与肺部微生物群高度相关^[14-16]^。肺部微生态与呼吸系统疾病之间存在密切关联，目前研究主要集中于肺部微生态与慢性阻塞性肺疾病、哮喘等慢性气道疾病之间的关联。但近些年来，越来越多的证据表明肺部微生物群与肺癌之间也存在密切关联。

### 1.1 肺癌患者具有特异的肺部微生物群

#### 1.1.1 肺部微生物群*α*多样性

Hosgood等^[17]^在中国宣威开展了一系列病例对照研究，最初研究纳入了8例不吸烟女性肺癌和8例健康人对照，对口腔和痰液两份标本进行测序，结果发现与健康对照组相比，肺癌患者的*Granulicatella*、*Abiotrophia*和*Streptococcus*属富集。之后其又纳入了45例肺癌和45例健康人对照，收集痰液标本进行测序。结果表明不吸烟人群中，肺癌风险的增加与微生物群*α*多样性较低有关^[18]^。其团队最新的一项巢氏病例对照研究发现，通过对114例肺癌患者和114例对照基线漱口水标本进行测序，结果表明微生物群*α*多样性较低的人群患肺癌的风险明显增高，但微生物群β多样性在病例组和对照组间未发现明显差异。进一步分析^[19]^发现，较高丰度的螺菌纲和类杆菌纲与肺癌风险降低有关，而较高丰度的杆菌类和乳酸菌类与肺癌风险增加有关。此外，Liu等^[20]^纳入24例肺癌患者和18例非肺癌患者，每个肺癌患者收集癌变部位和对侧非癌变部位的支气管刷检样本进行测序。结果发现从健康部位到非癌变部位到癌变部位，微生物群*α*多样性稳步下降；在属的层面上，链球菌和奈瑟菌属的丰度呈上升趋势，而葡萄球菌则逐渐下降。

与之相反的是，Greathouse等^[21]^纳入了143例肺癌病例和33例非肺癌对照，收集每例肺癌患者癌变部位和临近非癌变部位、非肺癌患者正常肺部的病理组织标本进行测序，结果表明，与非肿瘤邻近组织或肿瘤组织相比，在正常的肺部菌群*α*多样性较低。

#### 1.1.2 与肺部良性疾病患者微生物群的差异

Lee等^[22]^纳入了20例肺癌和8例良性肿块状病变患者，通过支气管镜收集了支气管肺泡灌洗液进行测序，结果表明肺癌患者和良性肿块状病变患者的肺部微生物群落存在显著差异，*Veillonella*属和*Megasphaera*属有可能作为预测肺癌的生物标志物。Liu等^[23]^发现肺癌患者的肺部微生物组成和群落结构与单纯肺气肿患者存在明显差异。他们纳入了10例肺气肿患者、11例肺癌患者和19例患者同时患有两种疾病的患者，收集患者肺部非肿瘤病理组织进行测序。结果发现，与仅肺气肿患者相比，肺癌患者肺部微生物组成的特点是变形杆菌（主要是*Acinetobacter*和*Acidovorax*属）的丰度明显较低，而类杆菌（链球菌）和类细菌（*Prevotella*）的感染率较高。

#### 1.1.3 肿瘤部位存在特异微生物群落

Yu等^[24]^收集165例肺癌患者的肿瘤部位和非肿瘤部位的病理组织，然后进行测序，结果发现肺部微生物群与口腔、鼻腔、粪便、皮肤和阴道中的微生物群落存在明显差异，其中变形杆菌是最主要的门类（60%）。Peters等^[25]^进行了一项19例非小细胞肺癌患者的预实验，收集了患者的肿瘤组织以及同一肺叶远端正常肺组织进行测序，结果发现肿瘤组织中微生物群落的丰富性和多样性低于配对的正常组织。

### 1.2 吸烟、室内燃煤和慢性支气管炎等因素可能会影响肺部微生物群落

Hosgood等^[19]^发现与使用无烟煤的病例相比，使用烟煤做饭和取暖的病例中，肺部微生物群的*α*多样性更高，主要是芽孢杆菌的差异。Yu等^[24]^研究表明肺部微生物*α*多样性随空气中的微粒暴露、地区人口密度从低到高以及吸烟包年数的增加而增加，但有慢性支气管炎病史的人群其肺部微生物*α*多样性减少。

### 1.3 病理类型影响肺部微生物群落多样性

Yan的研究^[26]^通过收集10例鳞癌，10例腺癌和20例对照的唾液标本进行测序，结果发现与对照组相比，鳞状细胞癌和腺癌患者唾液样本中的*Capnocytophaga*、*Selenomonas*、*Veillonella*和*Neisseria*属明显改变。肺鳞癌患者的微生物群落似乎比肺腺癌更多样化。Gomes等^[11]^纳入49例肺癌病例和54例非肺癌对照，收集支气管肺泡灌洗液进行测序，然后从The Cancer Genome Atlas选择509例腺癌和500例鳞癌来检测已经发现的微生物群。结果发现，肺癌微生物菌群富含变形杆菌，而且微生物群落比肺腺癌更多样化。蛋白菌群是肺癌的特异性菌群，其在肺鳞癌和肺腺癌中也被发现。肺鳞癌与一些肠杆菌科特异性相关。Greathouse等^[21]^在肺鳞癌中发现了单独的分类群，其中*Acidovorax*属在吸烟者中富集。此外，*Acidovorax*属在有TP53突变的鳞状细胞癌中表现出更高的丰度，而在腺癌中没有看到。但其他研究人员则得出了相反结论。Yu等^[24]^发现与鳞状细胞癌相比，腺癌中观察到较高的系统发育多样性，热力菌的相对丰度增加，拉氏菌的相对丰度减少。

### 1.4 肿瘤转移状态影响肺部微生物群多样性

Huang等^[27]^通过收集33例支气管灌洗液（14例鳞癌和19例腺癌）和52例痰液标本（15例鳞癌和37例腺癌）进行测序，结果发现无转移的肺腺癌中*Veillonell*、*Megasphaera*、*Actinomyces*和*Arthrobacter*属明显高于无转移的肺鳞癌。有转移的肺腺癌中*Capnocytophaga*和*Rothia*属的含量明显低于有转移的肺鳞癌。与无转移的肺腺癌相比，伴有转移的肺腺癌中链球菌属含量显著降低。*Veillonella*和*Rothia*属的含量在有转移的肺鳞癌中则明显高于无转移的肺鳞癌。

综上所述，肺部微生物群和肺癌之间的研究仍处于初步探索阶段，关于肺癌的认识远少于胃癌和结肠癌等其他癌症。目前，该领域研究仍有许多挑战有待解决：第一，已知危险因素的混杂作用，例如吸烟、暴露于各种职业致癌物（氡、砷、石棉等）、空气污染、肺癌家族史等危险因素。然而，目前几乎没有肺部微生物组的研究有足够样本量在多变量分析中调整上述已知的危险因素。第二，缺乏足够的对照来调整样本类型和环境污染物的系统偏倚。使用不同的标本类型，如肺部病理组织、痰液和支气管镜灌洗液等，它们对肺部微生物组的代表性可能存在差异。此外，对于微生物群落载量低的肺部样本，比如支气管灌洗液和肺组织活检，即使样本中较低的环境污染也可能对菌群测序结果产生较大影响。第三，需要长期纵向队列研究来阐明肺部微生物组和肺癌的因果关联，以及纳入不同时间点的样本来评估肺部微生物组的时序性变化，并且纳入不同国家/地区的人群来增加研究的代表性。

## 2 肺部微生物通过免疫反应诱导肺癌发生

宿主和微生物组之间的共生关系基于机体屏障和免疫系统^[28, 29]^。一旦屏障缺陷或免疫功能消失，就会发生微生物群的紊乱和细菌移位，导致微生物组与上皮细胞或免疫系统之间的病理性相互作用^[30, 31]^。

近年来研究揭示了肺部微生物组的变异在介导肺癌发生、发展中的作用。Jungnickel等^[32]^在慢性阻塞性肺疾病患者中发现，由非分型流感嗜血杆菌诱导的上皮细胞因子白介素17C（interleukin 17C, IL-17C），通过增加中性粒细胞炎症介导细菌的促肿瘤作用。Tsay等^[33]^发现，肺癌患者的下呼吸道富含口腔类群（链球菌和*Veillonella*属），这与ERK和PI3K信号传导途径的上调有关。体外实验结果也证明气道上皮细胞暴露于*Veillonella*、*Prevotella*属和链球菌，会产生相同的信号通路的上调。此外，微生物诱导的Th17细胞可促进肺癌细胞增殖和血管生成^[34]^。吸烟也可以通过诱导细菌因子的易位，进而促进肺癌细胞的转移生长和增殖^[35]^。共生菌群有助于小鼠模型中γδT17细胞对肺癌的应答^[36]^。

肺部微生物组与肺癌之间的机制研究仍处于初步阶段。目前大多数研究是在实验室小鼠中进行的，而人类与小鼠微生物群落结构的差异会导致该类研究外推的局限性。而且人类研究多为横断面研究，无法提供时间因果证据。此外，应该考虑rRNA测序技术的局限性，因为它不能区分活的和死的微生物。因此，宏基因组分析只是提供了微生物群落的组成，而没有提供微生物潜在的生物功能信息。宏基因组学、转录组学和代谢组学等多组学技术的结合对于研究肺部菌群与肺癌的机制关系至关重要^[37]^。

## 3 肺部微生物在免疫治疗中的作用

目前对微生物用于肺癌临床治疗应用的探索仍处于早期阶段，包括益生菌、饮食干预和粪便微生物群移植。了解人类肺部微生物群与肺癌之间的关系，可以为肺癌的诊断和治疗带来新的希望。

研究发现与恶性疾病相关的菌群失调或同时使用抗生素[免疫检查点抑制剂（checkpoint inhibitors, ICIs）治疗之前、期间或之后不久]可能影响ICIs疗效。在ICIs治疗期间，服用抗生素的非小细胞肺癌患者与没有接受抗生素治疗的患者相比，其无进展生存期和总生存期明显减少^[38]^。后续有研究比较了两组患者的肠道微生物群，并从康复患者的粪便中分离出了益生菌。而这种益生菌已被证明对预防肥胖症和糖尿病有效。此外，研究人员将康复患者的粪便植入无菌小鼠体内，那些接受“有效”粪便的小鼠对程序性死亡受体1（programmed cell death protein 1, PD-1）抑制剂的反应很快。此外，口服益生菌也能恢复免疫疗法的相同效果^[39]^。最近一项对接受免疫检查点抑制剂PD-1治疗的中国晚期非小细胞肺癌患者的研究表明，肠道微生物群多样性较高的患者对抗PD-1免疫检查点抑制剂的副反应更好。具有有利的肠道微生物群的患者（如多样性高的患者）在外周血^[40]^中表现出增强的记忆T细胞和自然杀伤细胞特征。

目前该领域的研究大多数集中在肠道微生物群，但抗生素也可能影响肺部微生物群，进而改变ICIs和其他免疫疗法对肺癌患者的抗肿瘤作用^[41]^。因此，肺部微生物组对免疫疗法的影响及其机制还需更多的探索。

## 4 小结

肺癌疾病负担严重，而肺部微生态的研究从新的角度认识肺癌。目前研究已初步证实肺癌患者存在肺部微生态的改变，而这些差异菌可能通过炎症反应、产生毒性代谢产物等机制参与肺癌的发生发展，并且跟肺癌的病理类型、肿瘤转移状态和预后等因素存在关联。因此，未来需要更多微生态的研究揭示肺部微生态与肺癌发生发展的因果关系，最终为临床诊断与治疗肺癌提供新的思路。
